# Investigating the Effects of Enzyme Inclusion Rates in Reduced Protein Diets to Improve Nutrient Digestibility in Laying Hens

**DOI:** 10.3390/ani16111713

**Published:** 2026-06-03

**Authors:** Aamir Nawab, Thi Hiep Dao, Sukirno Sukirno, Kenneth Bruerton, Eunjoo Kim, Tamsyn M. Crowley, Amy F. Moss

**Affiliations:** 1 School of Environmental and Rural Science, Faculty of Science, Agriculture, Business and Law, University of New England, Armidale, NSW 2351, Australia; 2 Faculty of Animal Science, Vietnam National University of Agriculture, Trau Quy Town, Gia Lam District, Hanoi 100000, Vietnam; 3 Faculty of Animal Science, University of Mataram, Jl. Majapahit 62 Mataram, Nusa Tenggara Barat, Mataram 83125, Indonesia; 4 Independent Researcher, Elanora, QLD 4221, Australia; 5 School of Life and Environmental Sciences, Faculty of Science, The University of Sydney, Camperdown, NSW 2006, Australia; 6 Poultry Research Foundation, The University of Sydney, Camden, NSW 2570, Australia; 7 Poultry Hub Australia, University of New England, Armidale, NSW 2351, Australia

**Keywords:** low protein diet, nutrient absorption, feed enzymes, nitrogen excretion

## Abstract

Enzymes have the potential to enhance nutrient utilisation and provide economic and environmental benefits in reduced protein (RP) diets; however, responses to different inclusion levels of phytase and carbohydrase enzymes remain inconsistent. Therefore, the present study aimed to evaluate the effects of phytase, xylanase, and β-glucanase inclusion levels in RP diets on nutrient digestibility and performance in laying hens. A total of 8 dietary treatments were allocated to 13 replicate cages of two hens per cage per treatment (n = 208) from 35 to 57 weeks of age. Experimental diets comprised two protein levels (standard protein (SP) at 16.5% crude protein versus RP at 14.5% crude protein), two phytase levels (600 and 1200 FTU/kg), and two XB levels (xylanase, β-glucanase 100 g/ton and 150 g/ton) were examined via a 2 × 2 × 2 factorial design. Between 35 and 57 weeks of age, no significant interactions were observed in laying performance parameters. However, RP diets significantly decreased egg weight across all periods, 35–47 weeks (*p* = 0.049), 48–57 weeks (*p* = 0.045), and overall, from 35 to 57 weeks (*p* = 0.044). Reduced protein diets also decreased egg mass from 35 to 47 weeks (*p* = 0.035). High Xylanase and β-glucanase (XB) inclusion (150 g/ton) lowered hen day egg production during 48–57 weeks (*p* = 0.015). At 47 weeks, RP diets reduced yolk weight (*p* = 0.021) and increased egg shape index (*p* = 0.048). Additionally, XB at 150 g/ton with 600 FTU/kg PhyG improved shell breaking strength (*p* = 0.046) but reduced it at 1200 FTU/kg. At 57 weeks, RP diets decreased albumen weight (*p* = 0.016), albumen proportion (*p* = 0.006) and increased yolk proportion (*p* = 0.005). Xylanase and β-glucanase inclusion at 150 g/ton reduced shell breaking strength (*p* = 0.036), and XB reduced yolk colour score (*p* = 0.038) only in SP diets. Also, RP diets reduced excreta moisture (*p* = 0.015) and improved dry matter digestibility (*p* = 0.020). Notably, feeding RP diets increased apparent metabolisable energy digestibility (*p* = 0.003), decreased protein excretion (*p* < 0.001) and increased apparent protein digestibility (*p* = 0.013). It is therefore concluded that decreasing RP diets to appropriate levels may benefit the environment without affecting the feed efficiency of laying hens.

## Simple Summary

The impact of enzyme supplementation, including phytase, xylanase, and β-glucanase, in reduced-protein (RP) diets for laying hens was investigated in this study. While there were no significant effects on laying performance, RP diets resulted in lower egg weight and egg mass during the study periods. High levels of xylanase and β-glucanase (150 g/ton) decreased egg production between 48 and 57 weeks, and reduced shell strength at certain points. However, a combination of xylanase, β-glucanase and phytase improved shell strength at a lower dose but had the opposite effect at a higher dose. Reduced protein diets also altered egg composition, reducing albumen weight and increasing yolk proportion. Notably, RP diets improved nutrient utilisation, decreased protein excretion and increased apparent metabolisable energy and protein digestibility. The study concludes that reducing protein levels in diets, when combined with appropriate enzyme supplementation, offers environmental benefits without compromising feed efficiency in laying hens.

## 1. Introduction

Reducing dietary crude protein (CP) in poultry diets has gained considerable attention due to its economic and environmental benefits, particularly through reducing feed costs and nitrogen excretion into the environment [1,2]. Interest in reduced-protein (RP) diets has further increased because of rising soybean meal prices and the need for more sustainable egg production systems. As feed represents the largest proportion of production costs in the livestock industry, including poultry [3]. Improving protein utilisation efficiency while maintaining performance is an important nutritional objective. Reduced protein diets supplemented with crystalline amino acids (AA) allow diets to more closely match the birds’ AA requirements and may improve nitrogen utilisation efficiency without compromising productivity [4]. However, because birds cannot synthesise essential AA endogenously [5]. So, careful balancing of limiting AA is critical during CP reduction.

Previous work from our research group demonstrated that, after lysine (Lys), methionine (Met), and threonine (Thr), valine (Val) becomes the fourth limiting AA, followed by tryptophan (Trp), isoleucine (Ile), arginine (Arg), and histidine (His) in wheat, sorghum, and soybean meal-based RP diets for laying hens [5]. Nevertheless, optimisation of crystalline AA alone may not fully overcome limitations associated with nutrient digestibility and anti-nutritional factors in RP diets. Phytate and non-starch polysaccharides (NSP) are major anti-nutritional components in cereal grains and soybean meal that can impair nutrient utilisation [6]. Phytate reduces phosphorus and AA availability through nutrient complex formation, whereas NSP increases intestinal viscosity and limits nutrient digestion and absorption. Consequently, exogenous enzymes such as phytase, xylanase, and β-glucanase are widely used in poultry nutrition to improve nutrient availability and digestive efficiency.

Phytase hydrolyses phytate complexes, increases phosphorus release, and improves AA and energy digestibility [7,8,9]. A systematic review by Cowieson et al. [9] reported improvements in the digestibility of Lys, Met, Thr, Val, Trp, Ile, Arg, and His following phytase supplementation in broiler diets. These responses may be particularly beneficial in RP diets in laying hens where AA availability is critical. However, RP formulations often contain lower soybean meal inclusion and higher wheat inclusion, which may reduce phytate substrate concentration and potentially alter phytase efficacy [10]. Similarly, xylanase and β-glucanase degrade NSP fractions, such as arabinoxylans and β-glucans in wheat-based diets, reduce digesta viscosity, and improve nutrient accessibility and intestinal function [11,12]. The reduction in intestinal viscosity may also enhance phytase access to phytate complexes by improving nutrient diffusion and enzyme-substrate interactions within the gastrointestinal tract. Therefore, combined supplementation of phytase and carbohydrase enzymes may exert complementary effects on nutrient digestion and absorption. However, studies evaluating xylanase and β-glucanase supplementation in laying hens have reported inconsistent responses [13,14,15,16,17], possibly due to differences in diet composition and NSP substrate concentration.

Importantly, RP diets may alter both phytate and NSP substrate availability because of changes in ingredient composition, particularly reduced soybean meal and increased wheat inclusion [10]. These changes may influence the efficacy and interaction of phytase, xylanase, and β-glucanase in laying hens. Despite the widespread use of these enzymes in poultry diets, limited information is available regarding their optimal inclusion rates and combined effects in RP diets during the post-peak laying period. Therefore, this study aimed to evaluate the effects of different inclusion levels of phytase and a carbohydrase complex containing xylanase and β-glucanase in RP diets for laying hens during the post-peak production phase. It was hypothesised that enzyme responses would differ between standard protein (SP) and RP diets due to differences in phytate and NSP substrate availability, and that combined enzyme supplementation would improve nutrient digestibility and nutrient utilisation efficiency in RP diets.

## 2. Materials and Methods

### 2.1. Birds and Animal Husbandry

This study was conducted at the Laureldale Research Station, University of New England. Animal ethics approval was obtained from the University of New England Animal Ethics Committee (ARA23-045), and all procedures complied with the Australian Code for the Care and Use of Animals for Scientific Purposes [18].

A total of 208 Hy-Line Brown pullets (15 weeks old) were obtained from a commercial layer farm in Tamworth, New South Wales, Australia. All birds originated from the same flock and were reared according to Hy-Line Brown management guidelines [19]. From 15 to 23 weeks of age, pullets were housed in barn-floor pens with 21 birds per pen. Subsequently, birds were transferred to a curtain-sided layer facility and housed in cages measuring 45 cm × 50 cm × 30 cm (height × depth × width), with two hens per cage. Between 24 and 28 weeks of age, hens participated in a separate 5-week enrichment study [20]. After completion of that study, hens remained in the same facility until the commencement of the present experiment at 35 weeks of age. Before the experimental period, all hens were fed a common commercial layer diet (Barastoc Premium Top Layer Mash; 16.5% CP, 2.5% crude fat, 6% crude fibre, 3.6% calcium, and 0.3% salt; Melbourne, Victoria, Australia). At 35 weeks of age, hens were individually weighed and randomly allocated to 104 cages (two birds per cage), ensuring similar mean body weights among treatments. Each cage was equipped with environmental enrichments, including a perch, scratch pad, and hanging CD, as described by Moss et al. [20]. Feed and water were provided ad libitum via one feed trough and two nipple drinkers per cage. Lighting was supplied using white LED bulbs (IP65 Dimmable LED Bulb, B-E27:10W, 5K) with a 16 h light: 8 h dark schedule controlled by an automatic timer. Ambient temperature and relative humidity inside the shed were monitored twice daily (morning and late afternoon) at bird height using a thermometer/hygrometer (Temp Alert, model R17HE910, S4GEM35XB, FCC RoHS compliant, 2011/65/EU, WI, USA). Throughout the experiment, shed temperature remained within acceptable ranges for laying hens, with average temperature ranges from approximately 18 to 27 °C and relative humidity of 40 to 73%. Upon completion of the experiment, hens were rehomed.

### 2.2. Experimental Design

The enzymes used in this study were phytase (PhyG; Axtra^®^ PHY Gold, Danisco Animal Nutrition, IFF, Marlborough, UK) and a carbohydrase enzyme containing xylanase and β-glucanase (XB; Axtra^®^ XB 201 TPT, Danisco Animal Nutrition, IFF, Marlborough, UK). A total of eight dietary treatments were arranged in a 2 × 2 × 2 factorial design, consisting of two protein levels (SP, 16.5% CP; RP, 14.5% CP), two PhyG levels (600 and 1200 FTU/kg), and two XB levels (100 g/ton: 1220 U/kg xylanase and 152 U/kg β-glucanase; 150 g/ton: 1830 U/kg xylanase and 228 U/kg β-glucanase). A total of 208 hens were allocated to 13 replicate cages per treatment, with two hens per cage. Each cage served as the experimental unit. The study was conducted over 23 weeks (35–57 weeks of age) in a layer cage facility to evaluate performance during the post-peak laying period when production and feed intake had stabilised. Initial body weights did not differ among treatment groups (*p* > 0.05).

### 2.3. Experimental Diets

Diets were formulated using wheat, sorghum, canola meal, barley, and soybean meal. The SP diet contained 16.5% CP according to Hy-Line Brown nutritional recommendations [21], while the RP diet was formulated with 14.5% CP (20 g/kg lower than SP). Essential AA levels were balanced based on Hy-Line Brown nutritional recommendations [21]. Enzyme inclusion levels for PhyG and XB were selected based on published recommendations [22] and manufacturer guidelines (Danisco Animal Nutrition, IFF). All diets were offered in mash form. Nutrient composition of main ingredients (dry matter (DM), apparent metabolisable energy (AMEn), CP, crude fat, crude fibre, AA, and minerals) was analysed using NIR spectroscopy (Foss NIR 6500, Hillerød, Denmark), calibrated with Evonik AMINONIR Advanced, and used for diet formulation [23]. The final diet compositions and calculated nutrient values are presented in Table 1. PhyG and XB were added to the RP diet at the expense of wheat to generate the remaining experimental diets. Analysed nutrient values of the final diets (DM, GE, CP, ash, Ca, P, and AA) were determined using standard methods [24] and are presented in Table 2. Overall, the analysed values closely matched the calculated values.

### 2.4. Data Collection

Data was collected over 23 weeks. Hens were weighed at 35, 47, and 57 weeks of age. Egg production and egg weight were recorded daily, and feed intake was recorded weekly. Egg mass and feed conversion ratio (FCR; kg feed/kg egg mass) were calculated from production, egg weight, and feed intake data. Egg quality was assessed at 47 and 57 weeks of age. At 57 weeks of age, excreta moisture and apparent digestibility of DM, metabolisable energy, and protein were determined using a total excreta collection method. Six hens per treatment (selected based on body weight close to the treatment mean) were used. Individual trays lined with aluminium foil were placed under cages to collect excreta. Feed residues and feathers were removed, and excreta were collected daily for 3 consecutive days (72 h; 9:00–13:00). Samples were thoroughly mixed, and sub-samples were aliquoted into 70 mL containers and stored at 4 °C until further analysis.

### 2.5. Egg Quality Analysis

A total of 100 and 104 eggs (approximately 13 eggs/treatment) were collected at 47 and 57 weeks of age, respectively, shortly after laying. Deformed eggs were excluded from the analysis. Egg length and width were measured using a digital Vernier calliper (Kincrome^®^, 0–150 mm range, Scoresby, Victoria, Australia) to calculate egg shape index (width/length). Shell reflectivity was measured using a shell reflectivity metre (Technical Services and Supplies, Dunnington, York, UK). Shell breaking strength and internal egg quality traits were assessed using a digital egg tester (DET6500^®^, Nabel Co., Ltd., Kyoto, Japan). Yolks were separated using Whatman filter paper and weighed individually. Albumen weight was calculated as the difference between whole egg weight and the combined yolk and shell weights. Eggshells were washed, air-dried for at least 72 h, and weighed using a precision balance (AdventurerTM, Model AX423, Ohaus, Parsippany, NJ, USA). Shell thickness, including the membrane, was measured using a Mitutoyo dial comparator gauge (Model 2109-10, Kawasaki, Japan). Egg component proportions were calculated as the percentage of albumen, yolk, and shell relative to total egg weight. All measurements were completed within 3 h of egg collection by trained personnel.

### 2.6. Wet Chemistry Analyses

Approximately 5 g of fresh excreta sample, collected at 57 weeks of age, was weighed into a pre-weighed crucible and dried in a forced air oven (Qualtex, Solidstat Temperature Control Oven, Model No. OM24SE3, Morningside, QLD, Australia) at 105 °C for approximately 48 h (to a constant weight), to determine DM content. The remaining portion of each excreta sample was preserved at −20 °C for further analysis. Frozen excreta samples were later subjected to freeze-drying using a freeze dryer (Christ Alpha 1-4 LD plus, Osterode am Harz, Germany). Both freeze-dried excreta and diet samples were ground into fine particles using an ultra-centrifugal mill (Retsch ZM 200, Fisher Scientific, Hampton, NH, USA) fitted with a 0.5 mm screen. Crude protein concentrations in the excreta and feed samples were measured using the Dumas combustion method [25] with a nitrogen analyser (LECO Corporation, St. Joseph, MI, USA), where EDTA was used as the calibration standard. Whereas GE levels in the excreta and feed samples were determined using a Parr Adiabatic Oxygen Bomb calorimeter (Parr Instrument Co., Moline, IL, USA), calibrated using benzoic acid. In addition, the ground freeze-dried excreta and feed samples were oven-dried at 105 °C for approximately 24 h (to a constant weight) to determine the DM content, following a method similar to that described for the fresh excreta samples. Apparent metabolisable energy and protein digestibility were calculated on a DM basis using equations described by Kong and Adeola [26].

Apparent protein digestibility (%) = (CPretained/CPintake) × 100

Apparent metabolisable energy digestibility (%) = (GEretained/GEintake) × 100

CPintake (g/day) = CPfeed (%) × FI (g/day/hen)

GEintake (kcal/day) = GEfeed (kcal/g) × FI (g/day/hen)

CPretained (g/day) = CPintake − CPexcreta (%) × excreta volume (g/day/hen)

GEretained (kcal/day) = GEintake − GEexcreta (kcal/g) × excreta volume (g/day/hen)

where CP, GE and FI are crude protein, gross energy, and feed intake, respectively.

### 2.7. Cost–Benefit Analysis

A cost–benefit analysis was conducted to evaluate the economic implications of dietary treatments using the following equation.

Feed cost per kilogram egg mass (AU$/kg egg) = Feed intake (kg) × Feed cost (AU$/kg)/Egg mass (kg)

### 2.8. Statistical Analysis

Data were organised and validated using Microsoft Excel, and statistical analyses were performed using IBM SPSS statistical software (Version 28.0.1.0; IBM Corp., Armonk, NY, USA). The data were analysed as a 2 × 2 × 2 factorial arrangement of dietary treatments using the univariate general linear model procedure using the SPSS^®^IBM Statistics 20 software programme (IBM Corporation, Somers, NY, USA). Main effects were determined using the independent samples *t*-test where appropriate. Prior to analysis, data were assessed for normality using the Kolmogorov–Smirnov test and for homogeneity of variances across treatment groups. The experimental unit was the cage mean, and statistical significance was declared at *p* ≤ 0.05.

## 3. Results

### 3.1. Laying Performance and Hen Weight

Table 3, Table 4 and Table 5 summarise laying performance at 35–47, 48–57, and 35–57 weeks of age, respectively. No significant protein × PhyG × XB or two-way interactions were observed for egg weight, hen-day egg production, egg mass, feed intake, or FCR across the experimental periods. Birds fed the RP diet showed lower egg weight during 35–47 weeks (*p* = 0.049), 48–57 weeks (*p* = 0.045), and over the entire study period (*p* = 0.044) compared with hens fed the SP diet. Reduced egg mass was also observed in hens fed the RP diet during 35–47 weeks of age (*p* = 0.035). Higher XB inclusion significantly reduced hen-day egg production during 48–57 weeks of age (*p* = 0.015). Mortality throughout the experiment was below 0.5% and was unrelated to dietary treatment. Hen body weight and weight gain at 35, 47, and 57 weeks of age was given in Table 6, where there was no significant effect of protein level, PhyG, or XB supplementation (*p* > 0.05).

### 3.2. Egg Quality

External egg quality at 47 weeks of age is presented in Table 7. Dietary protein level did not affect shell breaking strength, shell thickness, egg width, or shell reflectivity. However, hens fed RP diets had a higher egg shape index than those fed SP diets (*p* = 0.048). A significant PhyG × XB interaction was observed for shell breaking strength (*p* = 0.046), where increasing XB inclusion to 150 g/ton improved shell strength at 600 FTU/kg PhyG but reduced it at 1200 FTU/kg PhyG. Significant protein × XB and protein × PhyG × XB interactions were observed for egg width and length. XB supplementation reduced egg width in SP diets but increased it in RP diets (*p* = 0.041). In SP diets, adding 150 g/ton XB had no effect on egg length when combined with 600 FTU/kg PhyG, but it reduced egg length at 1200 FTU/kg PhyG. In contrast, in RP diets, 150 g/ton XB reduced egg length at 600 FTU/kg PhyG, while it increased egg length at 1200 FTU/kg PhyG.

Internal egg quality at 47 weeks of age (Table 8) was not affected by dietary treatments (*p* > 0.05).

Egg proportions at 47 weeks of age are shown in Table 9. Hens fed RP diets had lower yolk weight than those fed SP diets (*p* = 0.021). Significant protein × PhyG interactions were observed for albumen proportion (*p* = 0.032) and shell proportion (*p* = 0.044). Increasing PhyG from 600 to 1200 FTU/kg increased albumen proportion in SP diets but decreased it in RP diets. In contrast, increasing PhyG reduced shell proportion in SP diets but had no effect in RP diets.

External egg quality at 57 weeks of age is presented in Table 10. Increasing XB inclusion from 100 to 150 g/ton reduced shell breaking strength (*p* = 0.036) compared with the low level. No significant effects of dietary treatments were observed for shell thickness, egg dimensions, egg shape index, or reflectivity at 57 weeks of age.

Internal egg quality at 57 weeks of age is shown in Table 11. A significant protein × XB interaction was observed for yolk colour score (*p* = 0.038), where increasing XB reduced yolk colour in SP diets but had no effect in RP diets. No dietary effects were observed for albumen height, Haugh unit, yolk height, yolk diameter, or yolk index.

Egg proportions at 57 weeks of age are presented in Table 12. Hens fed RP diets had lower albumen weight (*p* = 0.016), higher yolk proportion (*p* = 0.005), and lower albumen proportion (*p* = 0.006) compared with hens fed SP diets. No treatment effects were observed for yolk weight, shell weight, or shell proportion.

### 3.3. Excreta Moisture Content and Apparent Nutrient Digestibility

Excreta moisture content and apparent total tract DM digestibility at 57 weeks of age are presented in Table 13. A significant main effect of dietary protein was observed, where RP diets reduced excreta moisture (*p* = 0.015) and improved DM digestibility (*p* = 0.020). Significant PhyG × XB interactions were detected for DM intake (*p* = 0.032) and excreted DM (*p* = 0.026), where increasing XB from 100 to 150 g/ton reduced DM intake and excretion at 600 FTU/kg PhyG, whereas the opposite response was observed at 1200 FTU/kg PhyG.

Apparent total tract metabolisable energy and protein digestibility at 57 weeks of age are presented in Table 14. A significant main effect of dietary protein level was observed, where RP diets increased metabolisable energy digestibility (*p* = 0.003), reduced protein excretion (*p* < 0.001), and improved protein digestibility (*p* = 0.013). Significant PhyG × XB interactions were observed for energy and protein intake. Increasing XB from 100 to 150 g/ton reduced energy and protein intake in diets with 600 FTU/kg PhyG, but increased energy (*p* = 0.017) and protein (*p* = 0.044) intake in diets with 1200 FTU/kg PhyG.

## 4. Discussion

In the present study, dietary CP levels were reduced through the inclusion of crystalline AA, resulting in an approximately 50% reduction in soybean meal content. While this strategy effectively reduced reliance on high-protein feed ingredients, it was associated with lower average egg weight and a tendency toward reduced egg mass across the experimental period. These results are consistent with Dao et al. [27], who also reported decreased egg weight in hens fed RP diets compared with SP diets. Similarly, Khajali et al. [28] observed reductions in egg mass and FCR in hens receiving RP diets between 40 and 44 weeks of age. Comparable responses have been widely reported, including decreases in egg weight, egg mass, and egg production under RP feeding systems [29,30,31]. These responses are likely linked to AA availability during albumen synthesis. The magnum, the primary site of albumen formation, deposits protein at approximately twice the rate of other oviduct segments [32]. As albumen production requires a continuous and high supply of AA, any limitation under RP diets may compromise synthesis efficiency, leading to reduced egg weight.

### 4.1. Laying Performance

In the current study, higher inclusion of XB significantly reduced egg production between 48 and 57 weeks of age. This response may be attributed to excessive xylanase activity, potentially generating elevated levels of xylo-oligosaccharides (XOS) through extensive degradation of both soluble and insoluble non-starch polysaccharides [33]. High concentrations of XOS can disrupt intestinal microbial balance and fermentation patterns [34] and may also increase gas production and impair nutrient absorption [35]. These effects may shift nutrient utilisation away from productive processes such as egg formation toward gut maintenance. This may explain the reduced performance observed at higher enzyme inclusion levels. Importantly, RP diets already contain lower NSP substrates due to reduced soybean meal inclusion, suggesting that the applied XB dose may have exceeded the requirement under these dietary conditions.

Previous studies have reported inconsistent responses to xylanase supplementation in laying hens. For instance, Mathlouthi et al. [14] found no improvement in egg production, egg weight, egg mass, or feed intake in hens fed xylanase (560 IU/kg) or β-glucanase (2800 IU/kg) diets. Similarly, Cufadar et al. [36] reported no significant effects of xylanase supplementation on performance or egg quality in wheat-based diets. Such variability in enzyme efficacy is largely attributed to differences in dietary substrate availability, enzyme dose, and formulation strategy. Bird age may also play a role, as digestive capacity and nutrient utilisation change over the laying cycle [37]. In contrast, positive performance responses have been reported in some studies where Xylanase or multi-enzyme complexes (xylanase, protease, and α-amylase) were used, particularly during early lay phases [38,39]. Overall, differences among studies likely reflect interactions among bird age, enzyme type and dose, formulation approach (with or without matrix values), and dietary composition.

### 4.2. Hen Weight

Previous studies have reported negative effects of RP diets on body weight in laying hens [29,31,40]. For example, Keshavarz [29] observed lower body weights in birds fed reduced protein levels (12 and 18% CP) during both the rearing and early laying periods compared with controls (12, 15, and 18% CP). In a subsequent phase (20–28 weeks), hens fed 18% CP gained more weight than those fed 14.5% or 16.5% CP, highlighting the sensitivity of growth responses to dietary protein level and production stage. Similarly, Alagawany et al. [31] reported that hens fed 18% of CP diet reached the final body weight of 1790 g, and a weight change of 139 g compared to those fed the RP diet (16%) from 18 to 34 weeks of age. Novak et al. [40] also reported reduced body weight in White Leghorn hens when dietary CP was lowered by approximately 3 percentage points, emphasising the importance of adequate protein for maintaining body weight during lay.

However, results across studies are not consistent. Moraes Sá et al. [41] found no significant effect of a 15% CP diet supplemented with methionine and cystine on body weight change in brown hens between 34 and 50 weeks of age. Similarly, Leeson and Caston [42] reported no adverse effects on body weight or feed intake when hens were fed a 14.4% CP diet from 40 to 70 weeks. Silversides et al. [43] further observed that phytase and xylanase supplementation did not influence body weight in Isa Brown hens at different production stages. In the present study, no significant effects of dietary protein level, PhyG, or XB supplementation were observed on body weight gain throughout the experimental period. Short- to mid-term feeding of RP diets may therefore have a limited impact on body weight when AA requirements are adequately met. However, prolonged feeding of RP diets, particularly when essential AA supply is marginal, may impair physiological functions such as egg formation, shell calcification, muscle maintenance, and immune competence. These processes depend on sufficient AA availability and energy balance. Therefore, long-term protein restriction without adequate AA supplementation may eventually compromise body weight and productivity, which may help explain the variability reported across studies.

### 4.3. Egg Quality

The present study showed that dietary treatments had no significant effects on external egg quality parameters, including eggshell thickness and shell strength at 47 and 57 weeks of age. These findings are consistent with previous reports. Kashani et al. [44] observed no differences in shell thickness or shell weight in hens fed RP diets (12.6–15% CP) at 59 and 63 weeks of age. Similarly, Oluwabiyi et al. [45] reported no effects of dietary protein level (14–18% and 16% CP) on external egg quality at 36 weeks of age. Gumpha et al. [46] also found that shell thickness was unaffected by CP levels ranging from 13% to 17.5%. In this study, however, hens fed 600 FTU/kg PhyG with 150 g/ton XB showed higher shell breaking strength at 47 weeks compared with those receiving 1200 FTU/kg PhyG with the same XB level. This aligns with reports showing improved eggshell quality with phytase supplementation [47,48,49]. For example, Kamińska [47] reported improved shell thickness and strength at 150 FTU/kg compared with 450 FTU/kg. Lim et al. [48] observed fewer shell defects at lower phytase inclusion levels, while Akyurek and Orhan [49] reported improvements in shell quality and shell thickness at 250 or 500 FTU/kg. Similarly, Dersjant-Li et al. [50] showed that 600 FTU/kg of phytase maintained shell quality comparable to higher inclusion levels (1200 FTU/kg). These responses suggest that excessive phytase inclusion without appropriate matrix adjustment may disrupt calcium-to-phosphorus (Ca:P) balance, potentially weakening shell quality. In the present study, enzymes were applied “over-the-top” without matrix values to avoid confounding effects in the factorial design. This approach may also explain the limited interaction effects observed.

Internal egg quality or egg component proportions were not affected by dietary treatments at either 47 or 57 weeks. This agrees with Novak et al. [51], who reported no significant effects of low-protein diets on wet and dry shell percentage or shell breaking strength. Similarly, Khajali et al. [52] found comparable egg quality in hens fed RP diets during both grower and peak production phases. In the present study, a higher phytase dose (1200 FTU/kg) reduced albumen proportion in RP diets at 47 weeks but increased it in SP diets. This opposite response may reflect differences in substrate availability between diets. de Lima et al. [53] also reported reduced egg solids, including albumen and yolk fractions, in hens fed low-protein diets (13% CP). Shell proportion was also unaffected by high phytase in RP diets but decreased in SP diets, consistent with findings by de Lima et al. [53], who reported minimal changes in egg component distribution under low-protein conditions with phytase supplementation. These results suggest that reduced substrate availability in RP diets may limit the responsiveness to higher enzyme inclusion. At 57 weeks, RP diets reduced albumen weight and percentage while increasing yolk proportion compared with SP diets in this study. Similar patterns have been reported in previous studies [32,51,54], where RP intake consistently decreased albumen deposition and increased yolk proportion.

These responses reflect the sensitivity of albumen synthesis to dietary protein supply. Albumen is primarily formed in the magnum region of the oviduct and is particularly dependent on AA availability during the early post-ovulatory phase [4]. Although crystalline AA may meet essential requirements, reduced total protein intake may limit substrate availability for optimal albumen deposition. In contrast, yolk deposition is more stable and less responsive to short-term dietary variation. Consequently, reduced albumen mass increases the relative proportion of yolk, thereby altering the internal egg composition. Overall, these findings highlight that not only AA balance, but also total dietary protein level is critical for maintaining optimal internal egg quality throughout the laying cycle.

### 4.4. Excreta Moisture Content and Apparent Nutrient Digestibility

This study demonstrated that dietary CP level influenced nutrient utilisation, particularly at 57 weeks of age. Hens fed RP diets showed lower excreta moisture content and higher apparent DM digestibility compared with those fed SP diets. Similar findings have been reported previously, where reduced CP diets improved DM and ether extract digestibility while decreasing excreta output and litter moisture [55,56]. The lower excreta moisture observed in RP-fed hens may be associated with reduced nitrogen intake and uric acid excretion, resulting in less water required for nitrogen elimination [55,57]. In contrast, excess dietary protein may increase nitrogen turnover and the amount of undigested protein reaching the hindgut, which can negatively affect gut microbial balance and nutrient utilisation [58,59,60,61]. These findings suggest that RP diets may support a more favourable digestive environment and improve nutrient efficiency in aged laying hens.

This study showed that supplementation with 600 FTU/kg PhyG and 150 g/ton XB reduced DM intake and excretion compared with 1200 FTU/kg PhyG at the same XB level; however, the differences were small and of limited practical relevance because DM digestibility was unaffected. Similarly, the combination of 600 FTU/kg PhyG and 150 g/ton XB reduced energy and protein intake at 57 weeks of age compared with the higher PhyG dose (1200 FTU/kg) with the same XB level. Previous studies have shown that increasing phytase supplementation can improve energy and nutrient digestibility [62,63,64,65]. Collectively, these results indicate that enzyme efficacy in RP diets depends on the balance between enzyme inclusion rate, nutrient matrix values, and dietary substrate availability. Reducing nitrogen excretion is important for improving the environmental sustainability of poultry production [66]. In the current study, RP diets significantly lowered nitrogen excretion while improving apparent metabolisable energy and protein digestibility at 57 weeks of age. Comparable reductions in nitrogen output from low CP diets have been reported previously in laying hens [1,40,67]. The reduction in nitrogen excretion may reflect both lower nitrogen intake and reduced protein fermentation in the hindgut, resulting in a more stable and beneficial gut environment [68,69]. These findings highlight the practical value of RP feeding strategies for improving nutrient efficiency while reducing the environmental impact of egg production.

## 5. Conclusions

Reducing dietary CP from 16.5% to 14.5% in laying hens decreased egg weight and altered some egg quality traits; however, feed efficiency was maintained. Importantly, RP diets reduced nitrogen excretion and excreta moisture while improving nutrient digestibility, indicating potential environmental and nutritional benefits. Supplementation with 600 FTU/kg PhyG and 100 g/ton XB was sufficient to support hen performance in RP diets from 35 to 57 weeks of age. Increasing XB inclusion to 150 g/ton did not provide additional benefits and was associated with reduced egg production, suggesting that enzyme responses in RP diets depend on substrate availability. Overall, these findings highlight the importance of optimising enzyme inclusion rates in RP diets to maintain production efficiency while improving nutrient utilisation and sustainability in laying hens. Future studies should evaluate appropriate matrix values and inclusion levels of PhyG and XB in RP diets with lower substrate availability to optimise hen performance without compromising egg quality or production.

## Figures and Tables

**Table 1:** Diet composition and calculated nutrient values of basal standard and reduced protein diets (as-fed basis).

Ingredients, g/kg	Standard Protein Diet	Reduced Protein Diet
Wheat	279.5	294.0
Sorghum	275	295
Soybean meal	162	88
Barley	100	125
Limestone grit	67	67
Canola meal	54	66
Fine limestone	30	30
Canola oil	22	17
Sodium bicarbonate	2.80	3.70
D,L-methionine	1.95	2.50
L-lysine HCl	1.05	3.10
L-threonine	0.10	1.00
Salt	1.70	1.00
Monocalcium phosphate	1.00	2.00
Choline Cl 60%	0.65	0.65
Xylanase ^1^	0.10	0.10
Phytase ^2^	0.06	0.06
Layer vitamin-mineral premix ^3^	1.00	1.00
Pigment Jabiru red	0.04	0.04
Pigment Jabiru yellow	0.03	0.03
L-valine	-	0.95
L-isoleucine	-	1.05
L-arginine	-	0.70
Total	1000	1000
Calculated nutrient (%, otherwise as indicated)		
AMEn ^4^, kcal/kg	2725	2725
Crude protein	16.5	14.5
Crude fat	4.10	3.70
Crude fibre	3.00	3.10
Ash	12.8	12.7
Total lysine	0.857	0.840
Total methionine	0.446	0.470
Total methionine + cysteine	0.738	0.734
Total threonine	0.623	0.610
Total isoleucine	0.656	0.642
Total leucine	1.342	1.150
Total tryptophan	0.225	0.190
Total arginine	0.973	0.840
Total histidine	0.393	0.330
Total valine	0.769	0.747
Digestible ^5^ lysine	0.735	0.735
Digestible methionine	0.421	0.450
Digestible methionine + cysteine	0.662	0.661
Digestible threonine	0.522	0.520
Digestible isoleucine	0.581	0.573
Digestible leucine	1.197	1.091
Digestible tryptophan	0.198	0.165
Digestible arginine	0.890	0.772
Digestible histidine	0.345	0.306
Digestible valine	0.660	0.647
Calcium	4.000	4.000
Available phosphorus	0.320	0.320
Total phosphorus	0.369	0.359
Sodium	0.180	0.180
Chloride	0.181	0.181
Potassium	0.633	0.569
Choline, mg/kg	1673	1590
Linoleic acid	1.400	1.300
Dietary electrolyte balance ^6^, mEq/kg	189	173

^1^ Axtra^®^ XB 201 TPT, Danisco Animal Nutrition, IFF, UK. ^2^ Axtra^®^ PHY Gold 10,000 FTU/g, Danisco Animal Nutrition, IFF, UK. ^3^ Vitamin-mineral premix (Rabar Pty Ltd., Beaudesert, Queensland, Australia) provided the following per kilogram diet: vitamin A, 10,000 IU; vitamin D, 3000 IU; vitamin E, 20 mg; vitamin K, 3 mg; nicotinic acid (niacin), 35 mg; pantothenic acid, 12 mg; folic acid, 1 mg; riboflavin (B2), 6 mg; cyanocobalamin (B12), 0.02 mg; biotin, 0.1 mg; pyridoxine (B6), 5 mg; thiamine (B1), 2 mg; copper, 8 mg as copper sulphate pentahydrate; cobalt, 0.2 mg as cobalt sulphate 21%; molybdenum, 0.5 mg as sodium molybdate; iodine, 1 mg as potassium iodide 68%; selenium, 0.3 mg as selenium 2%; iron, 60 mg as iron sulphate 30%; zinc, 60 mg as zinc sulphate 35%; manganese, 90 mg as manganous oxide 60%; antioxidant, 20 mg. ^4^ AMEn: N corrected apparent metabolizable energy. ^5^ Digestible amino acid coefficients of conventional feed ingredients were determined by Near-Infra Red spectroscopy (Foss NIR 6500, Hillerød, Denmark) standardised with Adisseo calibration. ^6^ Dietary electrolyte balance was calculated as 10,000 × (Na^+^ + K^+^ − Cl^−^).

**Table 2:** Analysed nutrient values of the experimental diets (as-fed basis) ^1^ and analysed values of phytase, xylanase and β-glucanase in experimental diets.

Factors	Experimental Diets							
Protein Level	Standard	Standard	Standard	Standard	Reduced	Reduced	Reduced	Reduced
Phytase gold (PhyG level; FTU/kg)	600	600	1200	1200	600	600	1200	1200
Xylanase and β-glucanase (XB level; g/ton)	100	150	100	150	100	150	100	150
Nutrient composition (%, otherwise as indicated)								
Dry matter	91.4	91.3	91.0	91.2	91.1	91.1	90.9	90.9
Gross energy (Kcal/kg)	3666	3656	3644	3707	3642	3664	3622	3658
Crude protein	16.0	16.1	16.3	16.5	14.2	14.1	14.5	14.6
Ash	13.2	13.2	12.8	12.1	12.3	12.1	12.6	12.2
Calcium	3.76	3.87	3.52	3.36	3.57	3.34	3.70	3.52
Total phosphorus	0.34	0.33	0.33	0.33	0.34	0.33	0.35	0.34
Aspartic acid	1.341	1.338	1.327	1.471	1.041	1.060	1.063	1.079
Serine	0.798	0.790	0.798	0.853	0.674	0.672	0.693	0.663
Glutamic Acid	3.260	3.267	3.223	3.551	2.918	2.989	2.984	3.037
Glycine	0.572	0.575	0.598	0.595	0.519	0.517	0.561	0.504
Histidine	0.380	0.376	0.391	0.402	0.335	0.343	0.365	0.335
Arginine	0.867	0.848	0.867	0.929	0.778	0.784	0.842	0.788
Threonine	0.563	0.565	0.575	0.606	0.586	0.573	0.629	0.584
Alanine	0.725	0.709	0.715	0.754	0.646	0.650	0.684	0.667
Proline	1.002	1.028	1.031	1.030	0.967	0.974	1.026	0.994
Tyrosine	0.424	0.447	0.446	0.467	0.399	0.400	0.442	0.417
Valine	0.509	0.513	0.546	0.605	0.580	0.589	0.662	0.629
Methionine	0.406	0.425	0.445	0.399	0.484	0.448	0.479	0.472
Lysine	0.822	0.819	0.820	0.899	0.834	0.832	0.856	0.874
Isoleucine	0.473	0.484	0.510	0.562	0.571	0.579	0.642	0.613
Leucine	1.173	1.175	1.205	1.256	1.061	1.076	1.169	1.119
Phenylalanine	0.726	0.754	0.762	0.786	0.679	0.681	0.734	0.697
Factors	Analysed levels							
PhyG level (FTU/kg)	1009	1296	1909	1745	987	1102	1920	1960
Xylanase U/kg	3987	5811	4034	4902	4785	5748	3786	5181
β-glucanase U/kg	241	285	251	291	231	288	240	251

^1^ Values of all the amino acids presented were total amino acids (measured on an as-is basis).

**Table 3:** Laying performance of hens fed the dietary treatments from weeks 35 to 47.

Effects			Egg Weight (g)	Hen Day Egg Production (%)	Egg Mass (g)	Feed Intake (g)	FCR (kg Feed/kg Egg)
Protein Level	PhyG Level (FTU/kg)	XB Level (g/Ton)					
Two-way interaction (PhyG × XB)							
	600	100	66.7	98.8	65.9	122	1.839
		150	66.0	98.6	65.1	123	1.867
	1200	100	65.8	98.1	64.1	119	1.864
		150	66.6	98.5	65.6	122	1.863
Three-way interaction							
Standard	600	100	67.0	98.6	66.0	123	1.837
		150	66.4	98.5	65.4	123	1.856
	1200	100	67.0	98.4	65.9	121	1.832
		150	67.1	98.0	65.8	121	1.849
Reduced	600	100	66.4	99.1	65.8	121	1.841
		150	65.6	98.6	64.7	124	1.879
	1200	100	64.7	97.8	62.3	117	1.896
		150	66.1	99.0	65.4	123	1.879
Main effect							
Protein	Standard		66.9 ^b^	98.4	65.8 ^b^	122	1.843
	Reduced		65.7 ^a^	98.7	64.5 ^a^	121	1.873
PhyG	600		66.4	98.7	65.5	123	1.853
	1200		66.2	98.3	64.8	121	1.864
XB	100		66.3	98.5	65.0	120	1.852
	150		66.3	98.5	65.3	123	1.865
Pooled SEM			0.31	0.16	0.30	0.79	0.009
*p*-values							
Main effect	Protein		0.049	0.405	0.035	0.643	0.103
	PhyG		0.810	0.220	0.256	0.203	0.548
	XB		0.950	0.848	0.581	0.177	0.445
Interactions	Protein × PhyG		0.405	0.833	0.179	0.877	0.365
	Protein × XB		0.652	0.393	0.234	0.282	0.813
	PhyG × XB		0.254	0.326	0.052	0.606	0.447
	Protein × PhyG × XB		0.543	0.131	0.112	0.743	0.476

^a,b^ Means within columns not sharing a common suffix are significantly different at the 5% level of probability. A standard protein diet contains 16.5% crude protein. Reduced protein diet contains 14.5% crude protein. PhyG = phytase Axtra^®^ PHY Gold (Danisco Animal Nutrition, IFF, UK) providing 10,000 FTU/g. PhyG supplemented at 60 g/ton and 120 g/ton provides 600 and 1200 FTU/kg, respectively. XB = Axtra^®^ XB 201 TPT (Danisco Animal Nutrition, IFF, UK). XB supplemented at 100 g/ton provides 1220 U/kg xylanase and 152 U/kg β-glucanase. XB supplemented at 150 g/ton to provide 1830 U/kg xylanase and 228 U/kg β-glucanase.

**Table 4:** Laying performance of hens fed the dietary treatments from weeks 48 to 57.

Effects			Egg Weight (g)	Hen Day Egg Production (%)	Egg Mass (g)	Feed Intake (g)	FCR (kg Feed/kg Egg)
Protein Level	PhyG Level (FTU/kg)	XB Level (g/Ton)					
Three-way interaction							
Standard	600	100	67.2	98.9	65.5	124	1.899
		150	66.6	97.0	64.6	122	1.900
	1200	100	67.5	98.4	66.4	123	1.857
		150	67.0	97.1	65.1	125	1.903
Reduced	600	100	66.2	98.5	65.2	122	1.874
		150	66.0	97.4	64.3	122	1.898
	1200	100	64.8	97.5	63.2	122	1.906
		150	66.3	97.9	64.9	125	1.928
Main effect							
Protein	Standard		67.1 ^b^	97.8	65.4	124	1.889
	Reduced		65.8 ^a^	97.8	64.4	122	1.901
PhyG	600		66.5	97.9	64.9	122	1.893
	1200		66.4	97.7	64.9	124	1.898
XB	100		66.4	98.3 ^b^	65.1	123	1.884
	150		66.5	97.4 ^a^	64.7	124	1.907
Pooled SEM			0.31	0.20	0.35	0.75	0.011
*p*-values							
Main effect	Protein		0.045	0.976	0.156	0.483	0.611
	PhyG		0.896	0.588	0.998	0.418	0.827
	XB		0.922	0.015	0.624	0.552	0.308
Interactions	Protein × PhyG		0.477	0.931	0.332	0.935	0.261
	Protein × XB		0.327	0.122	0.261	0.701	0.9996
	PhyG × XB		0.499	0.160	0.430	0.248	0.618
	Protein × PhyG × XB		0.515	0.603	0.291	0.901	0.608

^a,b^ Means within columns not sharing a common suffix are significantly different at the 5% level of probability. A standard protein diet contains 16.5% crude protein. Reduced protein diet contains 14.5% crude protein. PhyG = phytase Axtra^®^ PHY Gold (Danisco Animal Nutrition, IFF, UK) providing 10,000 FTU/g. PhyG supplemented at 60 g/ton and 120 g/ton provides 600 and 1200 FTU/kg, respectively. XB = Axtra^®^ XB 201 TPT (Danisco Animal Nutrition, IFF, UK). XB supplemented at 100 g/ton provides 1220 U/kg xylanase and 152 U/kg β-glucanase. XB supplemented at 150 g/ton to provide 1830 U/kg xylanase and 228 U/kg β-glucanase.

**Table 5:** Laying performance of hens fed the dietary treatments from weeks 35 to 57.

Effects			Egg Weight (g)	Hen Day Egg Production (%)	Egg Mass (g)	Feed Intake (g)	FCR (kg Feed/kg Egg)
Protein Level	PhyG Level (FTU/kg)	XB Level (g/Ton)					
Two-way interaction							
Protein × XB							
Standard	-	100	67.2	98.7	66.0	122	1.861
		150	66.8	97.7	65.3	123	1.882
Reduced	-	100	65.5	98.3	64.1	121	1.890
		150	66.0	98.3	64.9	123	1.892
PhyG × XB							
	600	100	66.7	98.9	65.7	123	1.875
		150	66.1	98.0	64.8	122	1.878
	1200	100	66.0	98.0	64.4	120	1.877
		150	66.6	98.1	65.3	123	1.896
Three-way interaction							
	600	100	67.1	99.0	65.8	123	1.880
		150	66.5	97.8	65.1	122	1.875
	1200	100	67.2	98.4	66.1	122	1.843
		150	67.1	97.6	65.5	123	1.888
Reduced	600	100	66.3	98.9	65.5	122	1.869
		150	65.8	98.1	64.6	123	1.880
	1200	100	64.7	97.7	62.7	119	1.911
		150	66.2	98.5	65.2	124	1.904
Main effect							
Protein	Standard		67.0 ^b^	98.2	65.6	122	1.871
	Reduced		65.7 ^a^	98.3	64.5	122	1.891
PhyG	600		66.4	98.4	65.2	123	1.876
	1200		66.3	98.1	64.9	122	1.886
XB	100		66.3	98.5	65.0	122	1.876
	150		66.4	98.0	65.1	123	1.887
Pooled SEM			0.30	0.15	0.31	0.73	0.010
*p*-values							
Main effect	Protein		0.044	0.750	0.069	0.780	0.336
	PhyG		0.845	0.188	0.543	0.689	0.624
	XB		0.937	0.120	0.959	0.398	0.578
Interactions	Protein × PhyG		0.429	0.929	0.230	0.768	0.269
	Protein × XB		0.486	0.094	0.232	0.404	0.650
	PhyG × XB		0.341	0.094	0.152	0.251	0.687
	Protein × PhyG × XB		0.524	0.319	0.169	0.832	0.414

^a,b^ Means within columns not sharing a common suffix are significantly different at the 5% level of probability. A standard protein diet contains 16.5% crude protein. Reduced protein diet contains 14.5% crude protein. PhyG = phytase Axtra^®^ PHY Gold (Danisco Animal Nutrition, IFF, UK) providing 10,000 FTU/g. PhyG supplemented at 60 g/ton and 120 g/ton provides 600 and 1200 FTU/kg, respectively. XB = Axtra^®^ XB 201 TPT (Danisco Animal Nutrition, IFF, UK). XB supplemented at 100 g/ton provides 1220 U/kg xylanase and 152 U/kg β-glucanase. XB supplemented at 150 g/ton to provide 1830 U/kg xylanase and 228 U/kg β-glucanase.

**Table 6:** Hen weight of the dietary treatments during the experimental period.

Effects			Hen Weight (g)			Weight Gain (g)		
Protein Level	PhyG Level (FTU/kg)	XB Level (g/Ton)	Week 35	Week 47	Week 57	Week 25–47	Week 47–57	Week 35–57
Three-way interaction								
Standard	600	100	2191	2346	2453	171	107	262
		150	2217	2407	2519	190	113	302
	1200	100	2186	2348	2425	162	115	239
		150	2197	2354	2489	166	125	291
Reduced	600	100	2214	2386	2505	172	119	291
		150	2218	2405	2505	191	123	286
	1200	100	2161	2328	2447	169	116	286
		150	2182	2385	2491	203	106	309
Main effect								
Protein	Standard		2198	2364	2472	172	115	273
	Reduced		2194	2375	2487	184	116	293
PhyG	600		2210	2385	2496	181	115	285
	1200		2182	2354	2463	175	115	281
XB	100		2188	2352	2458	169	114	269
	150		2204	2387	2501	188	116	297
Pooled SEM			12.67	14.18	16.89	6.03	3.95	7.94
*p*-values								
Main effect	Protein		0.868	0.667	0.659	0.340	0.884	0.228
	PhyG		0.274	0.263	0.346	0.606	0.988	0.786
	XB		0.550	0.216	0.211	0.126	0.798	0.076
Interactions	Protein × PhyG		0.539	0.802	0.927	0.393	0.224	0.420
	Protein × XB		0.915	0.926	0.531	0.538	0.518	0.244
	PhyG × XB		0.989	0.868	0.763	0.992	0.775	0.527
	Protein × PhyG × XB		0.761	0.425	0.739	0.571	0.562	0.806

A standard protein diet contains 16.5% crude protein. Reduced protein diet contains 14.5% crude protein. PhyG = phytase Axtra^®^ PHY Gold (Danisco Animal Nutrition, IFF, UK) providing 10,000 FTU/g. PhyG supplemented at 60 g/ton and 120 g/ton provides 600 and 1200 FTU/kg, respectively. XB = Axtra^®^ XB 201 TPT (Danisco Animal Nutrition, IFF, UK). XB supplemented at 100 g/ton provides 1220 U/kg xylanase and 152 U/kg β-glucanase. XB supplemented at 150 g/ton to provide 1830 U/kg xylanase and 228 U/kg β-glucanase.

**Table 7:** External egg quality of hens fed dietary treatments at week 47.

Effects			Shell Breaking Strength (kgf)	Shell Thickness (mm)	Egg Length (mm)	Egg Width (mm)	Egg Shape Index	Reflectivity (%)
Protein Level	PhyG Level (FTU/kg)	XB Level (g/Ton)						
Two-way interaction								
Protein × XB								
Standard	-	100	4.23	0.431	59.0	45.6 ^d^	0.777	24.9
		150	4.03	0.432	58.4	45.2 ^b^	0.778	24.9
Reduced	-	100	4.11	0.425	57.6	45.1 ^a^	0.783	25.2
		150	4.08	0.432	57.5	45.4 ^c^	0.789	26.5
PhyG × XB								
	600	100	4.07 ^b^	0.430	58.3	45.4	0.780	25.2
		150	4.24 ^c^	0.434	57.9	45.4	0.788	25.3
	1200	100	4.27 ^c^	0.425	58.4	45.3	0.780	24.9
		150	3.86 ^a^	0.430	58.0	45.2	0.779	26.3
Three-way interaction								
Standard	600	100	4.15	0.437	58.6 ^c^	45.7	0.781	25.0
		150	4.37	0.435	58.6 ^c^	45.2	0.778	24.4
	1200	100	4.32	0.425	59.4 ^d^	45.6	0.773	24.9
		150	3.59	0.427	58.0 ^b^	45.1	0.777	25.6
Reduced	600	100	3.99	0.424	58.0 ^b^	45.2	0.779	25.5
		150	4.12	0.432	57.1 ^a^	45.5	0.798	26.1
	1200	100	4.22	0.425	57.2 ^a^	45.0	0.788	24.8
		150	4.04	0.432	58.0 ^b^	45.2	0.780	26.9
Main effect								
Protein	Standard		4.14	0.431	58.7 ^b^	45.4	0.777 ^a^	24.9
	Reduced		4.09	0.429	57.6 ^a^	45.2	0.786 ^b^	25.8
PhyG	600		4.16	0.432	58.1	45.4	0.784	25.2
	1200		4.08	0.428	58.2	45.3	0.780	25.6
XB	100		4.17	0.428	58.3	45.4	0.780	25.0
	150		4.06	0.432	57.9	45.3	0.784	25.8
Pooled SEM			0.08	0.002	0.19	0.10	0.002	0.30
*p*-values								
Main effect	Protein		0.858	0.524	0.003	0.376	0.048	0.158
	PhyG		0.568	0.325	0.701	0.386	0.293	0.639
	XB		0.445	0.388	0.375	0.630	0.484	0.250
Interactions	Protein × PhyG		0.228	0.246	0.979	0.792	0.971	0.646
	Protein × XB		0.473	0.439	0.469	0.041	0.500	0.288
	PhyG × XB		0.046	0.877	0.888	0.787	0.249	0.267
	Protein × PhyG × XB		0.295	0.801	0.039	0.977	0.062	0.972

^a,b,c,d^ Means within columns not sharing a common suffix are significantly different at the 5% level of probability. A standard protein diet contains 16.5% crude protein. Reduced protein diet contains 14.5% crude protein. PhyG = phytase Axtra^®^ PHY Gold (Danisco Animal Nutrition, IFF, UK) providing 10,000 FTU/g. PhyG supplemented at 60 g/ton and 120 g/ton provides 600 and 1200 FTU/kg, respectively. XB = Axtra^®^ XB 201 TPT (Danisco Animal Nutrition, IFF, UK). XB supplemented at 100 g/ton provides 1220 U/kg xylanase and 152 U/kg β-glucanase. XB supplemented at 150 g/ton to provide 1830 U/kg xylanase and 228 U/kg β-glucanase.

**Table 8:** Internal egg quality of hens fed dietary treatments at week 47.

Effects			Albumen Height (mm)	Yolk Colour	Haugh Unit	Yolk Height (mm)	Yolk Diameter (mm)	Yolk Index
Protein Level	PhyG Level (FTU/kg)	XB Level (g/Ton)						
Two-way interaction								
Protein × PhyG								
Standard	600	-	9.50	11.1	94.8	23.2	35.6	0.666
	1200		8.87	11.5	91.1	22.6	38.1	0.616
Reduced	600	-	8.35	11.0	88.4	22.9	39.1	0.582
	1200		9.59	11.9	97.2	23.0	37.9	0.621
Protein × XB								
Standard	-	100	8.56	11.7	89.8	22.8	37.7	0.626
		150	9.91	10.9	96.8	23.1	35.9	0.659
Reduced	-	100	9.31	11.4	94.6	22.9	37.7	0.619
		150	8.53	11.4	90.8	22.9	39.4	0.582
Three-way interaction								
Standard	600	100	9.04	11.5	92.7	23.1	36.3	0.659
		150	9.97	10.8	97.0	23.4	35.0	0.674
	1200	100	8.12	11.8	86.6	22.6	38.9	0.596
		150	9.84	11.0	96.6	22.8	37.1	0.643
Reduced	600	100	8.96	11.0	90.7	22.8	38.4	0.602
		150	7.75	10.9	86.0	23.0	39.9	0.562
	1200	100	9.73	11.8	98.8	23.1	37.0	0.638
		150	9.45	12.0	95.5	22.9	38.9	0.603
Main effect								
Protein	Standard		9.19	11.3	93.1	23.0	36.9	0.641
	Reduced		8.92	11.4	92.7	22.9	38.5	0.601
PhyG	600		8.91	11.0	91.6	23.1	37.3	0.623
	1200		9.22	11.7	94.3	22.8	38.0	0.618
XB	100		8.93	11.5	92.2	22.9	37.7	0.623
	150		9.19	11.2	93.6	23.0	37.7	0.619
Pooled SEM			0.28	0.18	1.65	0.12	0.65	0.013
*p*-values								
Main effect	Protein		0.601	0.793	0.885	0.930	0.202	0.130
	PhyG		0.523	0.084	0.390	0.301	0.633	0.848
	XB		0.653	0.383	0.689	0.562	0.974	0.907
Interactions	Protein × PhyG		0.114	0.355	0.067	0.157	0.170	0.111
	Protein × XB		0.055	0.281	0.097	0.663	0.219	0.210
	PhyG × XB		0.432	0.918	0.597	0.569	0.979	0.736
	Protein × PhyG × XB		0.946	0.777	0.751	0.723	0.852	0.813

A standard protein diet contains 16.5% crude protein. Reduced protein diet contains 14.5% crude protein. PhyG = phytase Axtra^®^ PHY Gold (Danisco Animal Nutrition, IFF, UK) providing 10,000 FTU/g. PhyG supplemented at 60 g/ton and 120 g/ton provides 600 and 1200 FTU/kg, respectively. XB = Axtra^®^ XB 201 TPT (Danisco Animal Nutrition, IFF, UK). XB supplemented at 100 g/ton provides 1220 U/kg xylanase and 152 U/kg β-glucanase. XB supplemented at 150 g/ton to provide 1830 U/kg xylanase and 228 U/kg β-glucanase.

**Table 9:** Egg proportions of hens fed dietary treatments at week 47.

Effects			Yolk Weight (g)	Albumen Weight (g)	Shell Weight (g)	Yolk (%)	Albumen (%)	Shell (%)
Protein Level	PhyG Level (FTU/kg)	XB Level (g/Ton)						
Two-way interaction								
Protein × PhyG								
Standard	600	-	17.55	44.13	6.69	25.69	64.43 ^a^	9.86 ^c^
	1200		17.03	44.98	6.46	25.06	65.58 ^c^	9.42 ^a^
Reduced	600	-	16.74	44.26	6.47	24.96	65.59 ^c^	9.74 ^b^
	1200		16.76	42.63	6.45	25.34	65.19 ^b^	9.74 ^b^
Protein × XB								
Standard	-	100	17.37	45.20	6.66	25.10	65.21	9.69
		150	17.23	43.75	6.49	25.76	64.75	9.62
Reduced	-	100	16.56	43.03	6.44	25.17	65.52	9.77
		150	16.94	43.86	6.49	25.14	65.18	9.72
Three-way interaction								
Standard	600	100	17.49	44.52	6.74	25.45	64.63	9.93
		150	17.62	43.72	6.64	25.96	64.24	9.79
	1200	100	17.25	45.89	6.59	24.76	65.78	9.46
		150	16.76	43.79	6.28	25.50	65.38	9.36
Reduced	600	100	16.82	43.98	6.46	25.43	65.29	9.75
		150	16.65	44.58	6.49	24.45	65.89	9.74
	1200	100	16.28	41.90	6.42	24.92	65.79	9.78
		150	17.22	43.25	6.49	25.73	64.58	9.70
Main effect								
Protein	Standard		17.30 ^b^	44.54	6.58	25.40	65.00	9.66
	Reduced		16.75 ^a^	43.44	6.46	25.16	65.35	9.74
PhyG	600		17.14	44.19	6.58	25.34	64.99	9.80 ^b^
	1200		16.89	43.78	6.46	25.21	65.37	9.59 ^a^
XB	100		16.97	44.16	6.55	25.14	65.36	9.73
	150		17.08	43.81	6.49	25.43	64.98	9.67
Pooled SEM			0.12	0.35	0.05	0.17	0.19	0.05
*p*-values								
Main effect	Protein		0.021	0.130	0.232	0.413	0.289	0.382
	PhyG		0.290	0.511	0.162	0.777	0.310	0.045
	XB		0.619	0.755	0.456	0.415	0.338	0.459
Interactions	Protein × PhyG		0.268	0.079	0.218	0.187	0.032	0.044
	Protein × XB		0.249	0.090	0.176	0.291	0.881	0.758
	PhyG × XB		0.569	0.845	0.654	0.138	0.217	0.912
	Protein × PhyG × XB		0.072	0.465	0.506	0.262	0.226	0.788

^a,b,c^ Means within columns not sharing a common suffix are significantly different at the 5% level of probability. A standard protein diet contains 16.5% crude protein. Reduced protein diet contains 14.5% crude protein. PhyG = phytase Axtra^®^ PHY Gold (Danisco Animal Nutrition, IFF, UK) providing 10,000 FTU/g. PhyG supplemented at 60 g/ton and 120 g/ton provides 600 and 1200 FTU/kg, respectively. XB = Axtra^®^ XB 201 TPT (Danisco Animal Nutrition, IFF, UK). XB supplemented at 100 g/ton provides 1220 U/kg xylanase and 152 U/kg β-glucanase. XB supplemented at 150 g/ton to provide 1830 U/kg xylanase and 228 U/kg β-glucanase.

**Table 10:** External egg quality of hens fed dietary treatments at week 57.

Effects			Shell Breaking Strength (kgf)	Shell Thickness (mm)	Egg Length (mm)	Egg Width (mm)	Egg Shape Index	Reflectivity (%)
Protein Level	PhyG Level (FTU/kg)	XB Level (g/Ton)						
Three-way interaction								
Standard	600	100	3.99	0.447	58.5	45.6	0.780	23.6
		150	3.80	0.452	58.7	45.3	0.772	24.2
	1200	100	4.37	0.453	58.8	45.2	0.768	25.3
		150	3.44	0.441	58.8	44.9	0.765	24.3
Reduced	600	100	4.07	0.449	58.7	45.2	0.771	25.6
		150	4.08	0.439	58.3	45.3	0.778	24.7
	1200	100	4.30	0.449	57.6	44.9	0.779	24.3
		150	4.13	0.437	58.0	44.9	0.774	25.1
Main effect								
Protein	Standard		3.89	0.448	58.7	45.2	0.771	24.4
	Reduced		4.15	0.443	58.2	45.1	0.776	25.0
PhyG	600		3.99	0.447	58.5	45.4	0.775	24.5
	1200		4.05	0.445	58.3	45.0	0.772	24.8
XB	100		4.18 ^b^	0.449	58.4	45.2	0.775	24.7
	150		3.86 ^a^	0.442	58.4	45.1	0.772	24.6
Pooled SEM			0.08	0.002	0.17	0.10	0.002	0.23
*p*-values								
Main effect	Protein		0.093	0.270	0.131	0.459	0.291	0.201
	PhyG		0.608	0.725	0.491	0.058	0.370	0.559
	XB		0.036	0.139	0.870	0.574	0.543	0.799
Interactions	Protein × PhyG		0.639	0.844	0.205	0.999	0.145	0.137
	Protein × XB		0.105	0.408	0.897	0.455	0.443	0.849
	PhyG × XB		0.122	0.328	0.681	0.994	0.657	0.937
	Protein × PhyG × XB		0.350	0.412	0.512	0.760	0.305	0.085

^a,b^ Means within columns not sharing a common suffix are significantly different at the 5% level of probability. A standard protein diet contains 16.5% crude protein. Reduced protein diet contains 14.5% crude protein. PhyG = phytase Axtra^®^ PHY Gold (Danisco Animal Nutrition, IFF, UK) providing 10,000 FTU/g. PhyG supplemented at 60 g/ton and 120 g/ton provides 600 and 1200 FTU/kg, respectively. XB = Axtra^®^ XB 201 TPT (Danisco Animal Nutrition, IFF, UK). XB supplemented at 100 g/ton provides 1220 U/kg xylanase and 152 U/kg β-glucanase. XB supplemented at 150 g/ton to provide 1830 U/kg xylanase and 228 U/kg β-glucanase.

**Table 11:** Internal egg quality of hens fed dietary treatments at week 57.

Effects			Albumen Height (mm)	Yolk Colour	Haugh Unit	Yolk Height (mm)	Yolk Diameter (mm)	Yolk Index
Protein Level	PhyG Level (FTU/kg)	XB Level (g/Ton)						
Two-way interaction (Protein × XB)								
Standard	-	100	9.07	11.67 ^c^	92.0	22.9	41.9	0.546
		150	8.90	10.96 ^a^	91.1	22.9	40.9	0.550
Reduced	-	100	9.26	11.41 ^b^	94.0	23.2	40.7	0.574
		150	8.21	11.46 ^b^	87.8	23.1	42.1	0.551
Three-way interaction								
Standard	600	100	8.79	13.1	89.6	23.0	42.3	0.545
		150	9.23	11.8	92.3	23.1	41.7	0.533
	1200	100	9.37	12.3	94.6	22.8	41.5	0.548
		150	8.59	11.6	90.1	22.7	40.1	0.565
Reduced	600	100	9.51	11.3	95.3	23.2	40.1	0.582
		150	8.73	12.6	90.7	23.2	42.2	0.554
	1200	100	9.01	12.3	92.6	23.2	41.2	0.566
		150	7.73	12.0	85.2	23.0	41.9	0.549
Main effect								
Protein	Standard		8.99	12.2	91.6	22.9	41.3	0.548
	Reduced		8.75	12.0	90.9	23.2	41.3	0.564
PhyG	600		9.07	12.2	92.0	23.1	41.5	0.554
	1200		8.66	12.0	90.5	22.9	41.2	0.558
XB	100		9.16	12.2	93.0	23.1	41.3	0.561
	150		8.54	12.0	89.4	23.0	41.4	0.550
Pooled SEM			0.19	0.17	1.12	0.09	0.40	0.007
*p*-values								
Main effect	Protein		0.537	0.654	0.791	0.145	0.972	0.336
	PhyG		0.309	0.718	0.554	0.272	0.639	0.793
	XB		0.108	0.512	0.116	0.808	0.864	0.537
Interactions	Protein × PhyG		0.336	0.290	0.217	0.727	0.330	0.365
	Protein × XB		0.264	0.038	0.260	0.913	0.143	0.400
	PhyG × XB		0.267	0.442	0.272	0.588	0.508	0.514
	Protein × PhyG × XB		0.642	0.111	0.632	0.853	0.858	0.765

^a,b,c^ Means within columns not sharing a common suffix are significantly different at the 5% level of probability. A standard protein diet contains 16.5% crude protein. Reduced protein diet contains 14.5% crude protein. PhyG = phytase Axtra^®^ PHY Gold (Danisco Animal Nutrition, IFF, UK) providing 10,000 FTU/g. PhyG supplemented at 60 g/ton and 120 g/ton provides 600 and 1200 FTU/kg, respectively. XB = Axtra^®^ XB 201 TPT (Danisco Animal Nutrition, IFF, UK). XB supplemented at 100 g/ton provides 1220 U/kg xylanase and 152 U/kg β-glucanase. XB supplemented at 150 g/ton to provide 1830 U/kg xylanase and 228 U/kg β-glucanase.

**Table 12:** Egg proportions of hens fed dietary treatments at week 57.

Effects			Yolk Weight (g)	Albumen Weight (g)	Shell Weight (g)	Yolk (%)	Albumen (%)	Shell (%)
Protein Level	PhyG Level (FTU/kg)	XB Level (g/Ton)						
Three-way interaction								
Standard	600	100	17.30	43.38	6.83	25.69	64.18	10.17
		150	17.37	43.72	6.93	25.57	64.20	10.23
	1200	100	17.74	44.01	6.93	25.82	63.93	10.25
		150	17.18	42.92	6.68	25.77	64.20	10.16
Reduced	600	100	17.92	42.89	6.82	26.52	63.38	10.10
		150	17.87	42.20	6.75	26.79	63.10	10.11
	1200	100	17.35	41.04	6.78	26.67	62.96	10.42
		150	17.62	41.16	6.66	26.97	62.87	10.17
Main effect								
Protein	Standard		17.38	43.49 ^b^	6.84	25.71 ^a^	64.13 ^b^	10.20
	Reduced		17.70	41.84 ^a^	6.75	26.74 ^b^	63.08 ^a^	10.20
PhyG	600		17.62	43.04	6.84	26.15	63.70	10.15
	1200		17.46	42.24	6.76	26.32	63.48	10.25
XB	100		17.58	42.81	6.84	26.19	63.60	10.24
	150		17.51	42.50	6.76	26.27	63.59	10.17
Pooled SEM			0.12	0.34	0.05	0.19	0.19	0.06
*p*-values								
Main effect	Protein		0.213	0.016	0.325	0.005	0.006	0.979
	PhyG		0.534	0.246	0.413	0.659	0.563	0.414
	XB		0.810	0.632	0.379	0.780	0.951	0.540
Interactions	Protein × PhyG		0.309	0.339	0.972	0.997	0.795	0.442
	Protein × XB		0.485	0.963	0.894	0.617	0.671	0.663
	PhyG × XB		0.772	0.837	0.302	0.946	0.777	0.377
	Protein × PhyG × XB		0.343	0.416	0.424	0.978	0.968	0.814

^a,b^ Means within columns not sharing a common suffix are significantly different at the 5% level of probability. A standard protein diet contains 16.5% crude protein. Reduced protein diet contains 14.5% crude protein. PhyG = phytase Axtra^®^ PHY Gold (Danisco Animal Nutrition, IFF, UK) providing 10,000 FTU/g. PhyG supplemented at 60 g/ton and 120 g/ton provides 600 and 1200 FTU/kg, respectively. XB = Axtra^®^ XB 201 TPT (Danisco Animal Nutrition, IFF, UK). XB supplemented at 100 g/ton provides 1220 U/kg xylanase and 152 U/kg β-glucanase. XB supplemented at 150 g/ton to provide 1830 U/kg xylanase and 228 U/kg β-glucanase.

**Table 13:** Excreta moisture content and apparent dry matter digestibility of hens fed dietary treatments at week 57.

Effects			Excreta Moisture (%)	Dry Matter Intake (g/Day)	Dry Matter Excreted (g/Day)	Dry Matter Retained (g/Day)	Apparent Dry Matter Digestibility (%)
Protein Level	PhyG Level (FTU/kg)	XB Level (g/Ton)					
Two-way interaction (PhyG × XB)							
	600	100	76.5	115 ^c^	30.3 ^c^	84.3	73.6
		150	76.7	109 ^a^	27.6 ^a^	79.2	74.2
	1200	100	77.6	109 ^a^	28.9 ^b^	82.2	73.9
		150	77.0	113 ^b^	30.6 ^c^	81.9	72.8
Three-way interaction							
Standard	600	100	77.2	114	30.6	83.5	73.1
		150	77.8	106	27.7	78.5	74.0
	1200	100	77.6	110	30.4	80.0	72.5
		150	77.4	112	31.3	80.8	72.1
Reduced	600	100	75.9	115	29.9	85.1	74.0
		150	75.6	111	27.6	80.0	74.5
	1200	100	77.6	108	27.5	84.3	75.4
		150	76.6	113	29.9	83.0	73.5
Main effect							
Protein	Standard		77.5 ^b^	111	30.0	80.7	72.9 ^a^
	Reduced		76.4 ^a^	112	28.7	83.1	74.3 ^b^
PhyG	600		76.6	112	29.0	81.8	73.9
	1200		77.3	111	29.8	82.0	73.4
XB	100		77.1	112	29.6	83.2	73.8
	150		76.8	111	29.1	80.6	73.5
Pooled SEM			0.23	1.09	0.48	0.92	0.31
*p*-values							
Main effect	Protein		0.015	0.661	0.173	0.214	0.020
	PhyG		0.109	0.761	0.394	0.897	0.346
	XB		0.571	0.525	0.624	0.161	0.673
Interactions	Protein × PhyG		0.110	0.397	0.345	0.656	0.202
	Protein × XB		0.304	0.423	0.592	0.770	0.408
	PhyG × XB		0.365	0.032	0.026	0.203	0.124
	Protein × PhyG × XB		0.975	0.934	0.809	0.795	0.641

^a,b,c^ Means within columns not sharing a common suffix are significantly different at the 5% level of probability. A standard protein diet contains 16.5% crude protein. Reduced protein diet contains 14.5% crude protein. PhyG = phytase Axtra^®^ PHY Gold (Danisco Animal Nutrition, IFF, UK) providing 10,000 FTU/g. PhyG supplemented at 60 g/ton and 120 g/ton provides 600 and 1200 FTU/kg, respectively. XB = Axtra^®^ XB 201 TPT (Danisco Animal Nutrition, IFF, UK). XB supplemented at 100 g/ton provides 1220 U/kg xylanase and 152 U/kg β-glucanase. XB supplemented at 150 g/ton to provide 1830 U/kg xylanase and 228 U/kg β-glucanase.

**Table 14:** Apparent metabolisable energy and protein digestibility of hens fed dietary treatments at week 57.

Effects			Energy Intake per Day (kcal/Day)	Energy Excreted (kcal/Day)	Energy Retained (kcal/Day)	Apparent Metabolisable Energy Digestibility (%)	Protein Intake per Day (g/Day)	Protein Excreted (g/Day)	Protein Retained (g/Day)	Apparent Protein Digestibility (%)
Protein Level	PhyG Level (FTU/kg)	XB Level (g/Ton)								
Two-way interaction (PhyG × XB)										
	600	100	459 ^c^	97.4	362	78.8	18.9 ^b^	9.81	9.13	48.4
		150	435 ^a^	93.8	338	78.8	17.7 ^a^	9.31	8.37	47.5
	1200	100	437 ^a^	94.7	349	78.6	18.8 ^b^	9.40	9.43	50.1
		150	455 ^b^	98.4	355	77.9	19.2 ^c^	9.88	9.35	48.7
Three-way interaction										
Standard	600	100	458	100.4	357	78.0	20.0	11.1	8.81	44.2
		150	425	93.3	332	78.2	18.7	9.82	8.93	47.7
	1200	100	443	97.7	345	77.9	19.8	10.3	9.50	48.0
		150	456	99.2	353	77.4	20.3	11.0	9.33	45.9
Reduced	600	100	460	94.4	366	79.5	17.9	8.47	9.44	52.6
		150	447	94.4	343	79.4	16.6	8.80	7.82	47.3
	1200	100	430	91.8	353	79.3	17.8	8.48	9.36	52.1
		150	454	97.8	357	78.5	18.2	8.80	9.38	51.4
Main effect										
Protein	Standard		445	97.6	347	77.9 ^a^	19.7	10.6 ^b^	9.14	46.4 ^a^
	Reduced		449	94.6	355	79.2 ^b^	17.6	8.64 ^a^	9.00	50.9 ^b^
PhyG	600		447	95.7	350	78.8	18.3	9.56	8.75	47.9
	1200		446	96.5	352	78.3	19.0	9.64	9.39	49.4
XB	100		448	96.1	355	78.7	18.9	9.60	9.28	49.2
	150		446	96.1	346	78.4	18.5	9.59	8.86	48.1
Pooled SEM			4.41	1.30	3.84	0.23	0.25	0.22	0.20	0.91
*p*-values										
Main effect	Protein		0.802	0.240	0.317	0.003	< 0.001	< 0.001	0.723	0.013
	PhyG		0.882	0.767	0.764	0.271	0.078	0.825	0.114	0.419
	XB		0.788	0.983	0.244	0.431	0.297	0.984	0.303	0.503
Interactions	Protein × PhyG		0.294	0.836	0.829	0.885	0.956	0.841	0.812	0.815
	Protein × XB		0.394	0.282	0.956	0.734	0.888	0.364	0.337	0.294
	PhyG × XB		0.017	0.180	0.053	0.445	0.044	0.180	0.398	0.885
	Protein × PhyG × XB		0.821	0.805	0.816	0.982	0.975	0.175	0.231	0.148

^a,b,c^ Means within columns not sharing a common suffix are significantly different at the 5% level of probability. A standard protein diet contains 16.5% crude protein. Reduced protein diet contains 14.5% crude protein. PhyG = phytase Axtra^®^ PHY Gold (Danisco Animal Nutrition, IFF, UK) providing 10,000 FTU/g. PhyG supplemented at 60 g/ton and 120 g/ton provides 600 and 1200 FTU/kg, respectively. XB = Axtra^®^ XB 201 TPT (Danisco Animal Nutrition, IFF, UK). XB supplemented at 100 g/ton provides 1220 U/kg xylanase and 152 U/kg β-glucanase. XB supplemented at 150 g/ton to provide 1830 U/kg xylanase and 228 U/kg β-glucanase.

## Data Availability

The original contributions presented in this study are included in the article. Further inquiries can be directed to the corresponding authors.
